# Determinants of Quality of Life in Saudi Adults with Type 2 Diabetes Mellitus: A Cross-Sectional Study in Jeddah

**DOI:** 10.3390/healthcare14091228

**Published:** 2026-05-03

**Authors:** Amani A. Alrasheedi, Buthaina M. Aljehany

**Affiliations:** Food and Nutrition Department, Human Sciences and Design Faculty, King Abdulaziz University, Jeddah 22258, Saudi Arabia; aalrasheedi@kau.edu.sa

**Keywords:** quality of life, type 2 diabetes mellitus, obesity, dietary practices, glycemic control

## Abstract

Objective: This study aimed to assess quality of life (QoL) and its determinants among Saudi adults diagnosed with type 2 diabetes mellitus (T2DM). Methods: A cross-sectional study was conducted among 200 (45% male and 55% female) Saudi adults with T2DM aged 30–65 years. Data were collected using the Audit of Diabetes–Dependent Quality of Life (ADDQoL) and the Personal Diabetes Questionnaire (PDQ). Anthropometric and clinical measures included weight, height, body mass index (BMI), blood pressure, and glycated hemoglobin (HbA1c). Results: Most participants (73.5%) were ≤50 years of age, and the majority were obese (56.0%) or overweight (28.0%). Nearly half (54.5%) had HbA1c levels ≥ 8.0%, while (50.5%) were hypertensive. Overall, 96% of participants reported a poor to extremely poor QoL. Female sex (*p* = 0.003), higher BMI (*p* = 0.034), diet type (*p* = 0.039), and satisfaction with glucose control (*p* < 0.001) were significantly associated with the QoL. Conclusions: T2DM substantially impairs the QoL of affected Saudi adults. Psychosocial and lifestyle-related factors, particularly obesity, gender, dietary practices, and perceived glucose control, are more influential than traditional clinical markers. Culturally tailored interventions targeting these determinants may improve patient outcomes.

## 1. Introduction

Type 2 diabetes mellitus (T2DM) is a chronic metabolic disorder characterized by persistent hyperglycemia resulting from defects in insulin action [1]. Beyond metabolic dysregulation, T2DM represents a complex biopsychosocial condition that affects daily functioning, emotional well-being, and social participation [2,3], making it one of the fastest-growing global health challenges. In fact, according to the International Diabetes Federation (IDF), approximately 537 million adults worldwide were living with diabetes in 2021, and this number is projected to rise to 783 million by 2045 [4]. In the Middle East and North Africa (MENA) region, 73 million adults currently have diabetes, with the prevalence expected to increase by 87% by 2045 [4]. In Saudi Arabia, the burden of diabetes is particularly alarming, with an estimated national prevalence of 18.3%—placing the Kingdom among the top 10 countries worldwide for diabetes prevalence, according to the 2021 IDF statistics [4].

The etiology of T2DM is multifactorial, involving both non-modifiable risk factors such as age, genetics, and ethnicity and modifiable ones including dietary habits, physical inactivity, and smoking [5,6]. While clinical indicators such as HbA1c are essential for disease monitoring, they fail to fully capture patients’ lived experiences and perceived disease burden [7].

Beyond its clinical implications, diabetes exerts a profound influence on patients’ quality of life (QoL). QoL is a multidimensional construct encompassing physical, psychological, social, and environmental domains of well-being, as defined by the World Health Organization [8]. In chronic disease management, patient-reported outcomes such as their QoL are critical for evaluating holistic care effectiveness [9,10]. Patients with T2DM face multiple daily self-care demands, including medication adherence, dietary restrictions, and self-monitoring of blood glucose, all of which can adversely affect the QoL [2]. Studies have shown that poor glycemic control, obesity, diabetic complications, and comorbidities are consistently associated with a reduced QoL among patients with diabetes [7,11]. Conversely, effective self-management and social support can mitigate some of these negative effects [9].

In Saudi Arabia, evidence for the determinants of QoL among adults with T2DM remains limited, particularly in urban areas such as Jeddah. Previous regional studies have focused predominantly on glycemic outcomes, with limited exploration of the psychosocial and lifestyle determinants of QoL, particularly dietary behaviors and weight management practices [12,13]. Therefore, this study addresses this gap by assessing the quality of life of Saudi adults with T2DM in Jeddah and its associated factors, with particular emphasis on demographic, clinical, and lifestyle determinants, including dietary habits and weight management practices.

## 2. Methods

### 2.1. Study Design and Setting

A cross-sectional study was conducted with 200 adult Saudi patients diagnosed with T2DM, comprising 90 males and 110 females aged 30–65 years. Participants were recruited from the Department of Internal Medicine at King Abdulaziz University Hospital (KAUH), Jeddah, from September to December 2022. Data were collected using interviewer-administered questionnaires while patients were waiting for clinical consultation.

All participants were informed of the study objectives, and written consent was obtained prior to data collection. Ethical approval was granted by the Unit of Biomedical Ethics, the Research Ethics Committee, and the Faculty of Medicine Ethics Committee at King Abdulaziz University (No. 228-16) in 2016.

### 2.2. Study Procedure

Patients attending the Internal Medicine Department at King Abdulaziz University Hospital (KAUH) were approached consecutively during their routine clinic visits. The inclusion criteria were as follows: (1) Saudi nationality; (2) age 30–65 years; (3) confirmed diagnosis of T2DM for at least one year [10]; (4) ability to communicate in Arabic. The exclusion criteria were as follows: (1) presence of severe diabetic complications (e.g., end-stage renal disease, advanced retinopathy); (2) diagnosis of type 1 diabetes or gestational diabetes; (3) severe cognitive impairment or psychiatric illness; (4) pregnancy. Eligibility was confirmed based on the inclusion and exclusion criteria, after which participants were informed about the study objectives, and they provided written consent. Data collection was conducted through face-to-face interviews using the structured questionnaire, followed by anthropometric and clinical measurements carried out in a designated screening room to ensure consistency and accuracy.

A target sample size of 200 participants was calculated to achieve 80% statistical power to detect a small-to-moderate correlation (r = 0.20) between quality of life and key predictors at a two-tailed significance level of 0.05, accounting for an anticipated 10% non-response rate [14].

### 2.3. Study Tools

Quality of life (QoL) was assessed using the Audit of Diabetes–Dependent Quality of Life (ADDQoL) questionnaire [10], which is a diabetes-specific, patient-centered instrument widely used in international and Middle Eastern populations [12,15]. The instrument captures individualized QoL impacts by weighting domain importance, improving sensitivity to patient priorities compared with generic QoL tools.

The validated United Kingdom version was adapted to Arabic by Al-Shehri (2014), demonstrating adequate reliability and validity in the Saudi population [12]. The instrument evaluates the impact of diabetes across 19 life domains, along with two global items assessing overall QoL and diabetes-specific QoL. Items are rated on a 7-point scale (−3 to +3) for impact, and their importance is scored from 0 (not important) to 3 (very important).

The ADDQoL Average Weighted Impact (AWI) score ranges from −9 to +9, where more negative values indicate a poorer quality of life. Therefore, higher (less negative) scores represent a relatively better QoL.

In addition, the ADDQoL includes a global item assessing the overall quality of life, with response options ranging from “excellent” to “extremely bad.” For descriptive purposes, responses were categorized into “poor/extremely bad” and “fair/good.”

Selected items from the Personal Diabetes Questionnaire (PDQ) were used to assess behavioral and perceptual factors relevant to diabetes management, including satisfaction with glucose control, frequency of high blood sugar episodes, dietary practices, and weight loss attempts. These variables were analyzed as independent predictors of QoL. The PDQ was not used to generate a composite score; rather, individual items were analyzed for their clinical relevance [16].

A pilot study was conducted on 10 patients with diabetes to ensure the clarity and comprehension of the translated questionnaire; this was not included in the main analysis.

Operational Definition of Key Variables

Satisfaction with glucose control was assessed using a self-reported item from the Personal Diabetes Questionnaire (PDQ), with responses recorded on a Likert scale, where higher scores indicate higher satisfaction.

Frequency of high blood sugar episodes was assessed based on patient self-report and categorized according to the reported frequency (e.g., rarely, sometimes, often), with higher values indicating higher frequency.

Attempts to lose weight were assessed as a dichotomous variable (yes/no) based on participants’ self-reported efforts to reduce body weight.

Diet type was categorized as either following a structured dietary plan (e.g., medically prescribed or formal dietary program) or not following a structured plan.

### 2.4. Anthropometric and Clinical Measurements

- **Anthropometrics:** Weight was measured to the nearest 0.5 kg using a calibrated electronic scale (LAICA-LC76), LAICA S.p.A., Vicenza, Italy, and height to the nearest 0.1 cm using a Seca 213 Portable Stadiometer, Seca GmbH & Co. KG, Hamburg, Germany. Body mass index (BMI) was calculated as the weight (kg) divided by height (m^2^) and classified according to WHO guidelines [17] as follows: underweight (<18.5), normal (18.5–24.9), overweight (25.0–29.9), obese (≥30.0).
- **Blood Pressure (BP):** BP was measured and classified according to the American College of Cardiology/American Heart Association (ACC/AHA) 2017 guidelines as follows: normal (<120/<80 mmHg), elevated (120–129/<80 mmHg), stage 1 hypertension (130–139/80–89 mmHg), and stage 2 hypertension (≥140/≥90 mmHg) [18].
- **Glycosylated Hemoglobin (HbA1c):** Glycemic control was categorized as excellent (<6.5%), good (6.5–7.9%), fair (8.1–11%), and poor (≥8.0%) [19].

### 2.5. Statistical Analysis

Statistical analyses were performed using IBM SPSS Statistics version 26. Descriptive statistics (frequencies, percentages, means, and standard deviations) were used to summarize the sociodemographic, clinical, and QoL data. The ADDQoL AWI score was treated as a continuous variable for analysis. Pearson’s correlation and point-biserial correlations were used to examine the associations between QoL and continuous/dichotomous predictors, respectively. Independent *t*-tests and one-way ANOVA were used for group comparisons. Variables with *p* < 0.05 in univariate analyses were entered into a multiple linear regression model using the stepwise method to identify independent predictors of QoL. A stepwise multiple linear regression approach was used as an exploratory method to identify the potential predictors of QoL. This approach was chosen to balance model complexity and sample size; however, the results should be interpreted with caution. Assumptions of normality, homoscedasticity, and multicollinearity were checked and met. A *p*-value of <0.05 was considered statistically significant.

## 3. Results

### 3.1. General Characteristics of the Studied Population

A total of 200 patients with T2DM were enrolled in the study. The mean BMI of the participants was 31.56 ± 6.29 kg/m^2^. The median HbA1c category was 3.0 (IQR: 2.0–3.0), reflecting categorized glycemic control levels, and the mean systolic and diastolic blood pressures were 141.65 ± 20.81 mmHg and 75.04 ± 11.39 mmHg, respectively.

As shown in Table 1, more than half of the participants were female (55.0%) and under the age of 50 years (73.5%). The majority were married (75.0%), and more than half had a university-level education (56.0%). Over half of the participants were classified as obese (56.0%). The glycemic control was generally poor, with only 14.5% achieving excellent HbA1c levels, while 54.5% had poor control. Hypertension was present in 50.5% of participants. Importantly, based on the global ADDQoL item, 96% of participants rated their overall quality of life as poor or extremely bad.

### 3.2. Domain-Specific Quality of Life Scores and Predictions

The domain-specific analysis of the ADDQoL questionnaire revealed that the most negatively affected areas were health perception (−5.2 ± 1.5) and leisure activities (−4.5 ± 1.2), followed by social life (−3.5 ± 1.3) and work (−3.8 ± 1.4) (Table 2). Family life (−2.1 ± 1.0) was relatively less affected compared with other domains.

Correlation and regression analyses identified several significant predictors of the QoL (Table 3). Female sex, higher satisfaction with glucose control, and higher frequency of high blood sugar episodes were positively associated with higher (less negative) QoL scores, while higher BMI and attempts to lose weight were negatively associated. In the multivariate model, female sex (β = +0.18, *p* = 0.005), BMI (β = −0.12, *p* = 0.041), satisfaction with glucose control (β = +0.22, *p* < 0.001), attempts to lose weight (β = −0.14, *p* = 0.028), and high blood sugar frequency (β = +0.19, *p* = 0.006) remained independent predictors of the QoL, whereas age, HbA1c, and blood pressure were not significant predictors.

Group comparisons confirmed that QoL was significantly worse among patients with obesity compared with those of normal weight (*p* < 0.05), and participants following a structured diet plan reported a significantly better QoL compared with those not following a structured plan (*p* = 0.039) (Table 4). No significant differences in QoL were observed across HbA1c control categories or by hypertension status.

## 4. Discussion

T2DM has reached epidemic proportions globally and is also rapidly increasing in Saudi Arabia due to population growth, urbanization, sedentary lifestyles, and high rates of obesity and overweight [1,4,5,6]. In the present study, the overall quality of life (QoL) among T2DM patients in Jeddah was markedly poor, with 96% of participants reporting poor to extremely bad QoL. This proportion is substantially higher than that reported in Riyadh, where 46.5% of patients expressed very poor QoL [12], or in the UAE, where moderate QoL was more common [20]. These differences may reflect variations in lifestyle, cultural practices, healthcare access, or sample characteristics, but overall, they highlight the severe burden of diabetes on QoL in the Saudi population.

One of the strongest predictors of QoL in our study was gender, with female patients reporting significantly worse outcomes than males. This finding is consistent with previous Saudi studies [21,22] and international research [2,11], which consistently show that diabetic women have lower health-related QoL than men. Possible explanations include higher psychosocial stress, more caregiving responsibilities, and cultural expectations that may disproportionately affect women’s well-being. Obesity was also highly prevalent among our participants, and a higher BMI was independently associated with a poorer QoL, which aligns with findings from Bahijri et al. [13], who identified obesity as a major predictor of diabetes and prediabetes in Saudi Arabia, and with international studies linking obesity to an impaired QoL and higher complication risk [17,23]. The strong association between obesity and QoL emphasizes the urgent need for effective weight management strategies in Saudi diabetic populations.

Although HbA1c and blood pressure were not significant predictors of QoL in our study, patients’ satisfaction with their glucose control emerged as a strong independent predictor. A positive association was observed between frequency of high blood sugar episodes and QoL. Given that QoL scores in this study were predominantly negative, this finding indicates that individuals reporting more frequent hyperglycemic episodes had relatively better QoL scores, which appears counterintuitive and should be interpreted with caution. One possible explanation is that patients who are more engaged in self-monitoring and disease awareness may report both a higher frequency of hyperglycemic episodes and a better perceived quality of life. Alternatively, this association may reflect reporting bias or residual confounding factors not captured in the present analysis. Further longitudinal studies are needed to better understand the direction and nature of this relationship.

Overall, these findings suggest that subjective perceptions of control may have a stronger impact on the QoL than objective clinical measures. Previous studies have reported mixed findings, with some showing associations between HbA1c and QoL [10,24], while others highlighted the importance of patients’ self-efficacy and perceived control [7,25]. Our results support the latter, underlining the value of patient-centered care that focuses not only on clinical outcomes but also on patients’ lived experiences.

Dietary management was another key determinant of QoL. Patients who followed structured diet plans reported a significantly higher QoL compared to those without such plans. However, the adherence was very low, as only 6% of participants followed a formal food exchange system. This finding is concerning, given that diet is a cornerstone of T2DM management. Previous studies from Saudi Arabia have confirmed that many patients fail to adopt dietary recommendations, often due to cultural food habits, a lack of knowledge, or low motivation [26,27]. Structured dietary interventions, especially culturally adapted programs such as Mediterranean or low-GI diets, have shown significant improvements in glycemic control and patient well-being [4]. Our findings reinforce the need for targeted nutritional education and structured dietary support in Saudi diabetic care.

Interestingly, our results show that attempts to lose weight were associated with a lower QoL. This may reflect the psychological burden of repeated unsuccessful attempts, frustration with weight management, or increased awareness of one’s health limitations. Similar observations have been reported in other populations, where dieting without adequate support may negatively affect emotional well-being [28]. Therefore, comprehensive programs combining diet, exercise, behavioral therapy, and ongoing counseling may be essential to ensure that weight-loss efforts translate into an improved QoL.

Taken together, our findings highlight that QoL in Saudi T2DM patients is more strongly influenced by gender, BMI, diet adherence, weight control attempts, and perceived glucose satisfaction than by traditional clinical markers such as HbA1c or blood pressure. This highlights the need for clinical management approaches that extend beyond glycemic targets to address psychosocial and lifestyle factors. Interventions should therefore include structured lifestyle education, culturally tailored dietary guidance, and psychosocial support, particularly for women and obese patients.

The regression findings should be interpreted with caution. The use of a stepwise multiple linear regression approach, while useful for exploratory purposes, may be sensitive to sample-specific variations and data-driven variable selection. As such, the identified predictors should be considered as associated factors rather than definitive determinants of QoL. Future studies employing theory-driven modeling approaches and larger samples are recommended to confirm these findings.

To build upon the findings of this study, future research should prioritize longitudinal designs to establish causal relationships between identified predictors (e.g., dietary habits, weight loss attempts) and changes in QoL over time. Intervention studies are urgently needed to test the efficacy of integrated care models that combine nutritional counseling, psychological support, and diabetes education tailored for Saudi patients, with QoL as a primary outcome. Furthermore, research should explore the role of unmeasured variables, such as social support networks, health literacy, physical activity levels, and psychological distress (e.g., diabetes-specific distress, depression), which may mediate or moderate the relationships obtained in the present study. Qualitative investigations could provide deeper insight into the lived experiences of Saudi men and women with T2DM, particularly regarding barriers to dietary adherence and the emotional impact of weight management. Finally, multi-center studies across different regions of Saudi Arabia would help determine the generalizability of our findings and identify region-specific determinants of QoL.

This study has several limitations that should be acknowledged. Its cross-sectional design restricts the ability to establish causal relationships between the identified predictors and quality of life (QoL). The study was conducted at a single hospital in Jeddah, which may limit the generalizability of the findings to the broader Saudi population. In addition, several variables, such as dietary practices, weight control attempts, and satisfaction with glucose control, were based on self-reported data and are therefore subject to recall and social desirability bias. The sample size, although sufficient for the analyses performed, remains modest compared with larger multicenter studies.

Furthermore, important factors such as physical activity, detailed socioeconomic status, psychological comorbidities, the duration of T2DM, and diabetes-related complications were not assessed, although these variables may substantially influence QoL outcomes. Some observed associations, particularly those involving self-reported variables such as frequency of hyperglycemia, may also be influenced by reporting bias or subjective perception. Finally, the use of stepwise regression represents a methodological limitation, as this approach may lead to model instability and potential overfitting. Future research should employ theory-driven modeling approaches and larger and more diverse samples to validate these findings.

Practical Applications and Implications

The findings of this study may have implications for clinical practice and public health in Saudi Arabia. Routine assessment of QoL using validated instruments such as the ADDQoL may help identify patients at higher risk of impaired well-being. In addition, the results suggest that diabetes management approaches could benefit from moving beyond a purely glucocentric model toward a more holistic patient-centered approach.

Potential strategies may include the following:

- Providing targeted support services for female patients, such as peer support groups and gender-sensitive counseling.
- Incorporating psychological support and behavioral strategies into weight management programs to address the emotional impact of repeated weight control attempts.
- Enhancing healthcare provider training to include assessment of patients’ perceptions of glucose control and self-efficacy, in addition to traditional clinical indicators.

These approaches are consistent with the previous literature emphasizing the importance of psychosocial factors and patient-centered care in diabetes management [2,7].

## 5. Conclusions

This study highlights that the majority of Saudi adults with T2DM experience a markedly poor quality of life (QoL), with obesity, female gender, low adherence to a structured dietary plan, and dissatisfaction with glucose control emerging as significant determinants. In contrast, traditional clinical markers such as HbA1c and blood pressure are not strong predictors of QoL. These findings emphasize the importance of addressing psychosocial and lifestyle factors, in addition to clinical management, when caring for patients with diabetes.

To improve patient outcomes, culturally tailored nutritional education and structured dietary programs should be prioritized to support better adherence to healthy eating patterns. Special attention should also be given to weight management strategies, integrating diet, exercise, and behavioral counseling to reduce the burden of obesity and its impact on QoL. Furthermore, female patients may require targeted psychosocial and healthcare interventions to mitigate the disproportionate burden they face.

Healthcare providers are encouraged to adopt a patient-centered approach that considers not only glycemic targets but also patients’ perceptions, emotional well-being, and daily challenges. Future research should focus on longitudinal and interventional studies to evaluate the effectiveness of lifestyle interventions, psychological support, and culturally adapted care models in improving the QoL of Saudi diabetic populations.

## Figures and Tables

**Table 1:** Sociodemographic and clinical characteristics of participants (N = 200).

Variable	*n* (%)
**Sex**	
Male	90 (45.0)
Female	110 (55.0)
**Age group (years)**	
<40	65 (32.5)
40–49	82 (41.0)
≥50	53 (26.5)
**Marital status**	
Married	150 (75.0)
Unmarried	50 (25.0)
**Education**	
Primary/Secondary	88 (44.0)
University/Postgraduate	112 (56.0)
**Employment**	
Employed	95 (47.5)
Unemployed/Retired	105 (52.5)
**BMI category**	
Normal (<25)	32 (16.0)
Overweight (25–29.9)	56 (28.0)
Obese (≥30)	112 (56.0)
**HbA1c control**	
Excellent (<6.5%)	29 (14.5)
Good (6.5–7.9%)	62 (31.0)
Poor (≥8.0%)	109 (54.5)
**Hypertension**	
Normal	53(26.5)
Elevated	46 (23.0)
Stage 1 hypertension	51 (25.5)
Stage 2 hypertension	50 (25.0)
**QoL (overall)**	
Good	8 (4.0)
Bad	50 (25.0)
V. bad	37 (18.5)
E. bad	105 (52.5)

**Table 2:** Domain-specific quality of life scores (ADDQoL).

Domain	Mean ± SD	Range
Leisure activities	−4.5 ± 1.2	−9 to +3
Work and career	−3.8 ± 1.4	−9 to +3
Family life	−2.1 ± 1.0	−9 to +3
Health perception	−5.2 ± 1.5	−9 to +3
Social life	−3.5 ± 1.3	−9 to +3

**Table 3:** Correlation and regression analysis of predictors of QoL.

Variable	Univariate β (95% CI)	*p*-Value	Multivariate β (95% CI)	*p*-Value
Sex (female vs. male)	0.21 (0.07, 0.35)	0.003	0.18 (0.06, 0.30)	0.005
BMI (kg/m^2^)	−0.15 (−0.29, −0.01)	0.034	−0.12 (−0.24, −0.01)	0.041
Satisfaction with glucose control	0.25 (0.13, 0.37)	0.001	0.22 (0.11, 0.33)	0.001
Attempts to lose weight	−0.16 (−0.30, −0.02)	0.023	−0.14 (−0.27, −0.01)	0.028
High blood sugar frequency	0.23 (0.09, 0.37)	0.001	0.19 (0.05, 0.33)	0.006
Age	−0.08 (−0.22, 0.06)	0.250	—	—
HbA1c	−0.07 (−0.21, 0.07)	0.300	—	—
Blood pressure	−0.05 (−0.19, 0.09)	0.400	—	—

**Table 4:** Group comparisons of QoL scores (ANOVA/*t*-test).

Variable	Mean QoL ± SD	*p*-Value
BMI category		0.05 *
Normal (<25)	−2.8 ± 1.1	
Overweight (25–29.9)	−3.6 ± 1.3	
Obese (≥30)	−4.5 ± 1.4	
HbA1c category		0.06
Excellent (<6.5%)	−3.5 ± 1.2	
Good (6.5–7.9%)	−3.9 ± 1.3	
Poor (≥8.0%)	−4.2 ± 1.5	
Diet type		0.039 *
Structured plan	−3.1 ± 1.2	
No structured plan	−4.0 ± 1.4	
Hypertension		0.348
Normal	−3.5 ± 2.63	
Elevated	−4.33 ± 2.77	
Stage 1 hypertension	−4.30 ± 3.26	
Stage 2 hypertension	−3.10 ± 2.35	

* *p* ≤ 0.05 indicates statistical significance.

## Data Availability

Anonymized data from this study can be obtained from the corresponding author upon reasonable request.

## Abbreviations

ADDQoL	Audit of Diabetes–Dependent Quality of Life
BMI	Body Mass Index
DM	Diabetes Mellitus
GI	Glycemic Index
HbA1c	Glycated Hemoglobin
PDQ	Personal Diabetes Questionnaire
QoL	Quality of Life
T2DM	Type 2 Diabetes Mellitus
